# Reverse-genetics studies of lncRNAs—what we have learnt and paths forward

**DOI:** 10.1186/s13059-020-01994-5

**Published:** 2020-04-14

**Authors:** Fan Gao, Ye Cai, Philipp Kapranov, Dongyang Xu

**Affiliations:** Institute of Genomics, School of Biomedical Sciences, Huaqiao University, 201 Pan-Chinese S & T Building, 668 Jimei Road, Xiamen, 361021 China

## Abstract

Long non-coding RNAs (lncRNAs) represent a major fraction of the transcriptome in multicellular organisms. Although a handful of well-studied lncRNAs are broadly recognized as biologically meaningful, the fraction of such transcripts out of the entire collection of lncRNAs remains a subject of vigorous debate. Here we review the evidence for and against biological functionalities of lncRNAs and attempt to arrive at potential modes of lncRNA functionality that would reconcile the contradictory conclusions. Finally, we discuss different strategies of phenotypic analyses that could be used to investigate such modes of lncRNA functionality.

## Introduction

At the beginning of this century, the central dogma of biology that posits genetic information flow from DNA to RNA to protein was challenged by the discovery of pervasive transcription in the human genome [1, 2]. Long non-coding (lnc) RNAs account for most of the complexity of human transcriptome [3] and represent transcripts over 200 nucleotides in length [4] with no obvious protein-coding potential and a number of additional features (i.e., abundance, sequence conservation, splicing efficiency, subcellular localization, and others) that distinguish them from the canonical realm of protein-coding mRNAs [3, 5–7]. In the past decade, biological functions and molecular mechanisms of lncRNAs have attracted significant interest from the scientific community [8–12]. Although a number of lncRNAs have been associated with diverse biological processes and functions [13–15], for most part, these transcripts remain enigmatic. The most critical and yet the most controversial issue centers on biological significance of the lncRNA class of transcripts and the fraction of truly functional members it contains. Indeed, there is a growing body of contradictory evidence based on reverse-genetics assays that either supports or questions the broad biological functionality of this class of RNAs as described below. This leads to a great deal of confusion while also fueling the debate about functionality of these transcripts. One side in this debate argues that most of the currently annotated lncRNAs are not functional and represent spurious byproducts of mRNA biogenesis, leaky transcription, or other processes that confer no fitness advantage [16–20]. Consistent with these views, recent in vivo studies with knockouts of multiple lncRNAs reported no observable phenotypes [21–31]. Moreover, the biological functions of lncRNAs observed in different studies are often controversial, even with regard to some transcripts that are considered as the “gold standards” by the community (see below for details). Here, we will review these controversial observations and attempt to provide a theoretical framework that could potentially reconcile them. Finally, we will review emerging solutions based on the lessons learnt from previous reverse-genetics studies and novel experimental approaches that could answer the question of the biological relevance of lncRNAs.

**Table 1:** In vivo phenotypic studies of lncRNAs

lncRNA	Knockout strategy	In vivo phenotype	RNA-based rescue^1^	Phenotype not attributed to lncRNA^**2**^	Knockout technique^3^	Reference
*H19*	Replacement of a 3-kb gene region and 10 kb of 5′ flanking sequence of the lncRNA with a neomycin resistance cassette	Overgrowth in the animals inheriting the *H19* mutation from their mothers compared to those inheriting it from their fathers	N	Y^4^	HR	[32]
*H19*	Replacement of the entire lncRNA transcription unit with a *neo* cassette	Overgrowth (8%)	N	Y^5^	HR	[33]
*H19*	Same as above	Overgrowth in the lncRNA knockouts reflected in general (up to 20%) increase in weight. Corresponding decrease in weight was observed in knockout animals overexpressing the lncRNA.	Y		HR	[34]
*H19*	Same as in Ripoche et al. [33]. The E6.5 embryos were grafted into the wild-type or *Igf2*^*−/−*^ recipient mice to induce teratocarcinomas.	Increased weight of experimental teratocarcinomas	N		HR	[35]
	Knockout animals from Ripoche et al. [33] were bred with *Apc*^*∆14/+*^ mice	Increased number of adenomas compared with their *Apc* littermates	N		HR	
	Maternal heterozygotes of the *H19* knockout mice same as in Leighton et al. [32] were bred with *CRP-Tag 60-3* male mice.	Acceleration of liver tumor development	N		HR	
*H19*	Same as in Ripoche et al. [33]	Muscle hypertrophy and hyperplasia. A 50% reduction in the number of satellite cells	Y		HR	[36]
*H19*	Same as in Ripoche et al. [33]	Increased tumor development after carcinogen diethylnitrosamine treatment	N		HR	[37]
*H19*	Same as in Ripoche et al. [33]. The *H19* heterozygous male knockout mice were bred with the wild-type mice to generate paternal and maternal knockouts.	Increased liver weights immediately after birth	N		HR	[38]
*roX1/2*^6^	Deletion of the *roX2* gene, transposon insertion inactivation, or partial deletion of the *roX1* gene	Male-specific reduction in viability in the animals lacking both *roX1* and *roX2* genes	Y			[39]
*Xist*	Replacement of most of the lncRNA transcription unit with a *neo* cassette while leaving the promoter intact	Females carrying the *Xist* knockout on the paternal chromosome exhibited severe growth retardation and early embryonic lethality.	N		HR	[40]
*Xist*	Inversion of the exon 1 and deletion of the exon 4	Embryonic lethality in paternal knockout mice	N		HR and Cre	[41]
*Xist*	Mice with *loxP* sites inserted into *Xist* intron 3 and 5 kb upstream of the somatic cell promoter (*Xist*^*2lox/2lox*^) were bred with *Vav.Cre* mice to conditionally delete *Xist* in murine hematopoietic stem cells.	Females developed a highly aggressive myeloproliferative neoplasm and myelodysplastic syndrome with 100% penetrance.	N		Cre	[42]
*Xist*	The *Xist*^*fl/fl*^ or *Xist*^*∆/fl*^ mice generated using the same knockout strategy as above were crossed with *Sox2-Cre* mice to conditionally delete *Xist* in the epiblast lineage.	Females exhibited retarded growth, abnormal development of some organs, and failure to survive past weaning age.	N		Cre	[43]
*Xist*	*Xist*^*lox/lox*^ mice generated using the same knockout strategy as above were crossed with *MMTV-Cre* mice to generate animals with a mammary-specific knockout of *Xist.*	Acceleration of primary tumor growth in mammary glands and metastases in the brain	N		Cre	[44]
*Malat1*	Gene inactivation using insertion of the *lacZ* gene and polyadenylation signals immediately downstream of the transcriptional start site	No apparent phenotype	N/A		HR	[25]
*Malat1*	Same as above, but bred to *MMTV-PyMT* mice to generate *MMTVPyMT;Malat1*^*−/−*^ females	Promotion of lung metastasis in the knockout animals with breast cancer, contradictory to the results of Arun et al. (2016) [47]	Y		HR	[45]
*Malat1*	Deletion of a ~ 3-kb genomic region containing the 5′ end of the *Malat1* gene and its promoter	No apparent phenotype	N/A		HR, FLP, and Cre	[46]
*Malat1*	Same as Zhang et al. [46], but bred with *MMTV-PyMT* male mice	Reduction of branching morphogenesis in the *MMTV-PyMT* and *Her2/neu*-amplified tumor organoids, increase of cell adhesion, and loss of migration	N		HR	[47]
*Malat1*	Same as Zhang et al. [46]	Increased brain infarct size, worsened neurological scores, and reduced sensorimotor functions	N		HR	[48]
*Malat1*	Deletion of the complete 6982 bp *Malat1* transcript sequence plus 251 bp upstream of the *Malat1* transcription start site and 322 bp downstream of the *Malat1* transcript end	No apparent phenotype	N/A		HR and Cre	[23]
*Malat1*	Same as above	No apparent phenotype	N/A		HR and Cre	[24]
*Malat1*	Same as in Eissmann et al. [23], but crossed with *Apoe*^*−/−*^ mice	After a high-fat diet,the *Apoe*^*−/−*^*Malat1*^*−/−*^ mice showed increased plaque size and infiltration of inflammatory CD45+ cells, as well as enhanced adhesion of myeloid cells to atherosclerotic arteries compared to the *Apoe*^*−/−*^*Malat1*^*+/+*^ mice.	N		HR and Cre	[49]
*Hotair*	Deletion of the exons 1 and 2	Three notable anatomical phenotypes related to skeleton malformations	N		HR and Cre	[50]
*Hotair*	The same knockout strain as above, however, crossed into a different genetic background	No apparent phenotype attributable to the lncRNA, failure to reproduce the phenotypes above	N/A		HR and Cre	[26]
*Hotair*	Replacement of a 2.3-kb genomic sequence from exon 1 to the last annotated exon with a *lacZ*-neomycin resistance cassette	Morphological malformations in caudal vertebra	N		HR	[51]
*Neat1*	Gene inactivation using insertion of the *lacZ* gene and polyadenylation signals immediately downstream of the transcriptional start site	No apparent phenotypes except for disappearance of paraspeckles	N/A		HR	[52]
*Neat1*	Same as above	Stochastic failure to become pregnant in a subpopulation of the knockout animals	N		HR	[53]
*Neat1*	Presumably the same as in Nakagawa et al. [52]	Aberrant mammary gland morphogenesis and lactation defects	N			[54]
*Neat1*	Same as in Nakagawa et al. [52]	Preneoplastic cells were sensitized to DNA-damage-induced cell death, and skin tumorigenesis was impaired.	N		HR	[55]
*Neat1*	Same as in Nakagawa et al. [52]	Decrease of neointima formation following vascular injury	N		HR	[56]
*Neat1*	Deletion of the entire lncRNA transcription unit	Reduction of inflammatory responses	N		Cas9	[57]
*Fendrr*	Replacement of exon 1 with a transcriptional stop signal (3x pA)	Embryonic lethality and impairment of the heart and body wall	Y		HR	[58]
*Firre*	Deletion of the entire *Firre* gene body and promoter	Cell-specific hematopoietic phenotypes	Y		HR and Cre	[59]
*lncKdm2b*	Deletion of a 838-bp fragment containing the exon 2	Impaired embryonic stem cell self-renewal and early embryonic lethality	Y		Cas9	[60]
*PCFL*	Deletion of a 6475-bp region containing *PCFL* and adjacent sequences	Improved heart function and reduced cardiac fibrosis after myocardial infarction in heterozygous knockout animals	Y		Cas9	[61]
*Chaer*	Deletion of exon 2	Attenuated cardiac hypertrophy and blunted pathological fibrosis following trans-aortic constriction	N (in vitro rescue only)		Cas9	[62]
*linc1405*	Deletion of exon 2	Impaired heart development and function	N (in vitro rescue only)		Cas9	[63]
*lincRNA-EPS*	Replacement of the entire 4-kb genomic locus with a neomycin cassette	Enhanced inflammation and lethality following endotoxin challenge	N (in vitro rescue only)		HR	[64]
*lncKdm2b*	Same as in Ye et al. [60]	Early embryonic lethality. Impaired intestinal group 3 innate lymphoid cell (ILC3) maintenance and proliferation	N (in vitro rescue only)		Cas9	[65]
	Mice with *loxP* elements flanking the exon 2 of *lncKdm2b* were crossed with *Vav-Cre*^*+*^ mice to generate animals with a conditional deletion of *lncKdm2b* from the bone marrow.	Markedly decreased absolute numbers of ILC3s	N		Cas9 and Cre	
	Mice with *loxP* elements flanking the exon 2 of *lncKdm2b* were crossed with *Rorc-Cre*^*+*^mice to generate mice with conditional deletion of *lncKdm2b* from ILC3s.	Remarkably decreased numbers of all ILC3 subpopulations	N		Cas9 and Cre	
*ANRIL*	Deletion of the 70-kb region on Chr 4 containing the mouse gene aligning to human 58-kb non-coding CAD risk interval	Showed a protective effect on diabetic mouse kidneys (lowering of urine volume and urine albumin levels in comparison with the wild-type diabetic animals)	N		HR and Cre	[66]
*Blnc1*	Adipose tissue-specific deletion of the entire gene	Mice with fat-specific inactivation of *Blnc1* showed impaired cold-induced thermogenesis and browning and exacerbation of obesity-associated brown fat whitening, adipose tissue inflammation, and fibrosis, leading to a more severe insulin resistance and hepatic steatosis.	N		Cas9 and Cre	[67]
*Blnc1*	Whole body deletion of the entire gene	Liver X receptor agonist-induced rise in plasma triglyceride and hepatic steatosis was significantly blunted by *Blnc1* deficiency.	N		Cas9	[68]
	Liver-specific deletion of the entire gene	Abrogation of high-fat diet-induced hepatic steatosis and insulin resistance and prevention of diet-induced nonalcoholic steatohepatitis				
*Bmncr*	Deletion of the 4.92-kb sequence of *Bmncr*	Decreased bone mass and increased bone marrow adiposity	N		HR	[69]
*βlinc1*	Replacement of the *βlinc1* sequence with the *puΔtk-EM7-kan* cassette	Defective islet development and glucose-intolerance in the adult mice	N		HR	[70]
*Charme*	Insertion of a polyadenylation/MAZ cassette into the beginning of the exon 2	Peculiar heart remodeling phenotype (changes in size, structure, and shape of the organ), morphological alteration of skeletal and cardiac muscles, and shortened lifespan	N		Cas9 and HR	[71]
*CPR*	Replacement of a 2968-bp fragment of *CPR* gene containing exons 1 and 2 with a *neo* cassette	Restored heart function after myocardial injury (increased cardiomyocyte proliferation, improved myocardial function, and reduced scar formation)	N		HR	[72]
*Cyrano*	Deletion of the first half of the exon 3	No overt abnormalities	N/A		HR and Cre	[27]
*Dino*	Replacement of the bulk of Dino sequence with GFP	Dampened *p53* signaling and ameliorated acute radiation syndrome	N		HR	[73]
	Inactivation of promoter					
*Evf2*	Insertion of a triple polyadenylation transcription stop site into the exon 1	No apparent phenotype, except for reduced numbers of GABAergic interneurons in early postnatal hippocampus and dentate gyrus	N		HR	[74]
*Fendrr*	Replacement with a *lacZ* reporter cassette	Perinatal lethal and lung, heart, and gastrointestinal tract defects	N		HR	[75]
*Fendrr*	Replacement of a 19-kb genomic sequence from the exon 2 to the last annotated exon with a *lacZ*-neomycin resistance cassette	Perinatal lethal	N		HR	[51]
*Flatr*	Deletion of the promoter region and the majority of the exon 1	No reported in vivo phenotype	N		Cas9 and HR	[76]
	Deletion of the entirety of the exon 2				Cas9	
*Flicr*	Deletion of the whole exon 2	*Flicr*-deficient non-obese diabetic female mice showed a significantly reduced rate and incidence of overt diabetes.	N		Cas9	[77]
*Gm26878*	A 2.3-kb deletion involving the entire lncRNA-encoding gene	Neonatal lethal with low penetrance	N		Cas9	[78]
*Gomafu*	Deletion of the entire lncRNA gene (157 kb)	Hyperactive behavior with increased sensitivity to the psychostimulant methamphetamine	N		HR and Cre	[79]
*Gtl2/Meg3*	Replacement of the exons 1–5 (10 kb) with a *neo* cassette	Maternal knockout pups died within 4 weeks after birth. Paternal knockout mice showed severe growth retardation and perinatal lethality. Homozygous mutants survived and developed into fertile adults.	N		HR	[80]
*Gtl2/Meg3*	Replacement of the first five exons and adjacent upstream promoter sequences of ~ 300 bp with a *neo* cassette	Perinatal death and skeletal muscle defects in the mice with the maternal deletion	N		HR	[81]
*Gtl2/Meg3*	Same as above	Skeletal muscle defects and perinatal death in the maternal knockout animals, as well as increased microvessel formation in the brain	N		HR	[82]
*Gtl2/Meg3*	Same as above	Increased microvessel formation in the brain	N		HR	[83]
*Hottip*	Replacement of the 4.8-kb genomic sequence from the exon 1 to the last annotated exon with a *lacZ*-neomycin resistance cassette	Gastrocnemius muscle defects and hindlimb skeletal malformation	N		HR	[51]
*Linc-Brn1b*	Replacement with a *lacZ* reporter cassette	Growth defects (reduced number of intermediate progenitor cells in the cerebral cortex, abnormal cortical lamination and disorganization of the barrel cortex, reduced body weight)	N		HR	[75]
*Linc-pint*	Replacement with a *lacZ* reporter cassette	Growth defects (noticeably smaller and reduced body weight)	N		HR	[75]
*Linc-pint*	Replacement of the 32-kb genomic sequence from the exon 2 to the last annotated exon with a *lacZ*-neomycin resistance cassette	Growth deficiency (slower growth rate, age-dependent abnormal hindlimb clasping posture, fur loss, lower fat content and femur bone mineral density, decreased muscle mass, and lordokyphosis)	N		HR	[51]
*Linc-RAM*	Deletion of the exon 2	Delayed muscle regeneration	N		HR and Cre	[84]
*lincRNA-p21*	Mice with *loxP* sites flanking the *p53* response element in the promoter and exon 1 of the lncRNA were crossed with the *Deletor Cre* mice to achieve a conditional knockout.	No significant abnormalities	N/A		HR, FLP and Cre	[28]
*LncDACH1*	*LncDACH1*^*Flox/Flox*^ mice were crossed with α-myosin heavy chain *Cre* mice to generate mice with a cardiac myocyte-specific knockout of *LncDACH1.*	Increased calcium transient, cell shortening, and improved cardiac function of transverse aortic constriction induced heart failure mice.	N		Cas9 and Cre	[85]
*lncGata6*	Deletion of the region from the exon 2 to the exon 4	Impaired stemness of intestinal stem cells (ISCs) and intestinal regeneration	N		Cas9	[86]
	Insertion of an SV40 poly(A) (STOP) module into the promoter of the lncRNA	Same as above				
	Mutation in the lncRNA exon 4	Same as above				
	Insertion of *loxP* sequences flanking the exons 2–4 of the *lncGata6* locus and establishing *Lgr5*^*GFP-CreERT2*^; *Rosa26*^*lsl-lacZ*^; *lncGata6*^*f/f*^ mice	Reduction of ISCs with suppressed cycling and proliferation of ISCs compared to *Lgr5*^*GFP-CreERT2*^; *Rosa26*^*lsl-lacZ*^ mice			Cas9 and Cre	
*Lnc-mg*	Conditional deletion of the exon 1 in the muscle	Muscle atrophy and the loss of muscular endurance during exercise	N		HR and Cre	[87]
*lncOb*	Deletion of the 5′ end of the lncRNA first exon	Increased fat mass with reduced plasma leptin levels and lost weight after a leptin treatment	N		Cas9	[88]
*lncRNA-155*	Deletion of most of the lncRNA sequence	Increased susceptibility to influenza A virus infection	N		HR	[89]
*Mdgt*	Replacement with a *lacZ* reporter cassette	Reduced viability and growth	N		HR	[75]
*PEAT*	Deletion of the entire lncRNA transcribed unit	No apparent phenotype	N/A		Cas9	[29]
*Peril*	Replacement with a *lacZ* reporter cassette	Reduction of viability, death shortly after birth as well as reduced body weight	N		HR	[75]
*Redrum*	Deletion of the lncRNA exon 3	No apparent phenotype	N/A		Cas9 and HR	[30]
*Rik-201 and Rik-203*	Deletion from the beginning of second exon to the end of the third exon of the lncRNA C130071C03Rik	Abnormal brain development	N		Cas9	[90]
*Silc1*	Deletion of the lncRNA promoter and exon 1	Delayed regeneration of sensory neurons following injury	N		Cas9	[91]
*SRA*	Insertion of a *lacZ/neo* cassette with transcription termination signals before the exon 3	Obesity resistance and improved glucose tolerance in knockout mice fed a high-fat diet	N		HR	[92]
*SYISL*	Deletion of a 1133-bp genomic region containing most of the *SYISL* transcript	Increased muscle fiber density, muscle mass, and regeneration	N		Cas9	[93]
*Tsix*	Deletion of a 3.7-kb CpG-rich domain at the 5′ end of *Tsix* that included the putative promoter and transcriptional start site	The knockout mice showed normal paternal but impaired maternal transmission. Maternal inheritance is infrequent, with surviving progeny showing intrauterine growth retardation and reduced fertility.	N		HR	[94]
*Tsix*	Insertion of an IRES*βgeo* cassette in the second exon to disrupted lncRNA transcripts from both promoters	Inheritance of the disrupted maternal allele resulted in ectopic *Xist* expression and early embryonic lethality	N		HR	[95]
*Tslrn1*	Deletion of the entire lncRNA transcribed region	Male knockout mice displayed normal fertility but a significant reduction in spermatozoa.	N		Cas9	[96]
*Tsx*	Deletion of a 2.1-kb region encompassing the predicted promoter region, exon 1, and 160 bp of intron 1	Male mutant animals have smaller testes and altered behavior with less fear and enhanced short-term memory.	N		HR, FLP and Cre	[97]
*Visc-2*	Deletion of the entire lncRNA locus	No overt anatomical or behavioral phenotype	N/A		HR	[31]
*Air/Airn*	Insertion of a polyadenylation cassette to truncate *Air* to 4% of its length	Mice with the maternally inherited mutant allele were identical to the wild type. Animals with the paternally inherited mutant allele or homozygous mutant mice showed a 15% reduction in birth weight.	N	Y^7^	HR and Cre	[98]
*Crnde*	Ablation of the whole coding region	Low bone mass phenotype due to impaired osteoblast proliferation and differentiation	N (in vitro rescue only)	Possible^8^	Cas9	[99]
*Hand2as/Hand2os1/lncHand2/Uph*	Deletion of the exon 1 and/or exon 2	Liver damage and liver regeneration defects	Y		Cas9	[100]
	Conditional deletion of the exon 2 in hepatocytes	Severe liver injury, much poorer liver regeneration capacity, and a smaller liver mass	N		Cas9 and Cre	
*Hand2as/Hand2os1/lncHand2/Uph*	Insertion of a triple polyadenylation sequence into the exon 2	Right ventricular hypoplasia and embryonic lethality	N	Y^9^	TALENs	[101]
*Hand2as/Hand2os1/lncHand2/Uph*	Deletion of the entire lncRNA locus	Septum lesion, heart hypoplasia, and perinatal death	N	Y^10^	Cas9	[21]
	Deletion of a 2.7-kb DNA sequence that spans exons 4 and 5	Severe contraction defects in adult heart that progressively worsened with increasing age				
	Deletion of the 5′ promoter and first two exons	No discernable heart phenotypes in either embryos or adults				

^1^*Y* yes, *N* no, *N/A* not applicable

^2^*Y* yes

^3^*HR* homologous recombination, *Cre* Cre-mediated recombination, *Cas9* CRISPR/Cas9, *FLP* FLP-mediated recombination

^4^The phenotype of the *H19* knockout mice was attributed to a gain of function of *Igf2* due to the loss of a common imprinting control element caused by the *H19* deletion instead of the deletion of the *H19* gene itself

^5^The phenotype was also attributed to the increase in the *Igf2* expression via deletion of a shared imprinting control element mapped to a 10-kb region upstream of *H19*

^6^All studies were done in mouse with the exception of the *roX* genes done in *Drosophila melanogaster*

^7^Latos et al. [102] (see the text) reported that Airn transcriptional overlap, but not its lncRNA products, induces imprinted *Igf2r* silencing

^8^Szafron et al. [103] (see the text) showed that CRNDE encodes a nuclear peptide (CRNDEP) which may be involved in the regulation of the cell proliferation

^9^The phenotype was caused by blockade of the lncRNA transcription, but not the knockdown of the mature transcript

^10^The DNA locus, rather than its transcription/transcripts, was shown to be primarily responsible for the heart development and function phenotypes

## Evidence supporting the biological roles of lncRNAs

**Fig. 1:**
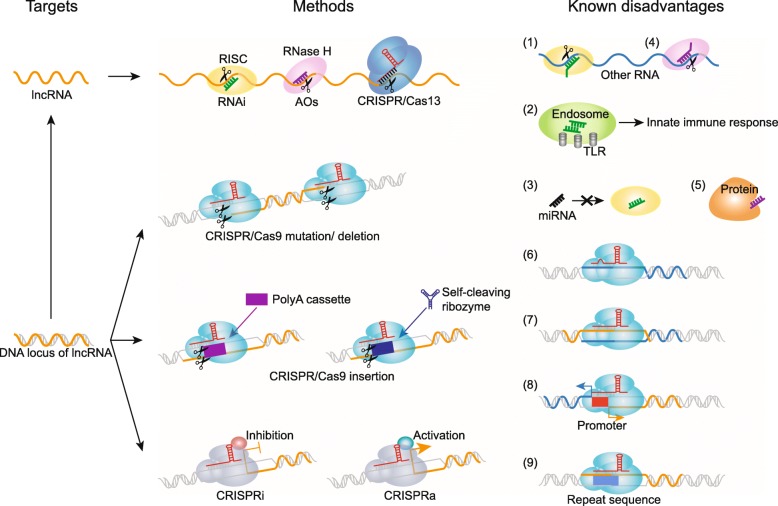
Reverse-genetics approaches for lncRNA functional studies. The illustration shows various methods that target either RNA (based on RNAi, ASOs, or CRISPR/Cas13) or DNA, based on the CRISPR/Cas9 family of methods that can cause deletions and insertions of specific sequences (e.g., polyA cassettes or self-cleaving ribozymes) or bring transcription activators/silencers to promoters depending on specific system employed. Also shown are some of the known problems with these techniques—off-target effects caused by partial sequence matches (1, 4, 6) or non-specific effects such as triggering innate immune response (2), saturation of the endogenous RNAi machinery (3), and interactions with proteins (5), as well inability to discriminate between the targets and other overlapping (7) or shared elements (8) and to target sequences containing repetitive elements (9). More details are in the text

Ample body of research based on a variety of techniques supports the notion that lncRNAs do play biological roles in a variety of biological processes. Below, we attempt to review and summarize the main outcomes of these studies in the context of the different reverse-genetics (Fig. 1) and evolutionary approaches employed. While in this review we focus on techniques that directly address biological relevance of lncRNAs, clues to their biological importance can also be gleaned from understanding their mechanisms of function at the molecular level and therefore we would like to direct the reader to several reviews published recently on this topic [8–11, 13–15, 104, 105].

### RNA targeting

Methods that specifically target an RNA molecule without altering its DNA sequence or transcription represent the most direct means of answering the question of the functionality of an lncRNA transcript per se without the potential confounding effects caused by disruption of an important DNA element or hampering progression of the RNA polymerase complex (see below) (Fig. 1). Phenotypic assays based on depletion of lncRNAs using two such technologies—RNAi or antisense oligos (ASOs)—represent perhaps the majority of the empirical evidence supporting the functionality of these transcripts. Indeed, both RNAi, either based on transfected siRNAs or endogenously expressed shRNAs, and ASOs have been used widely to show phenotypic consequences of depleting various types of lncRNAs. Below, we provide examples of such studies for individual transcripts that represent various types of lncRNAs that could be differentiated based on the form of the apparent functional product, e.g., spliced or unspliced and polyadenylated or non-polyadenylated, and subcellular localization (nuclear or cytosolic).

Knockdown of a nuclear unspliced polyA+ lncRNA *Neat1* by RNAi led to ablation of paraspeckles, suggesting the essential role of this lncRNA in the formation of these subnuclear compartments [106]. Knockdown of an unspliced, polyA− nuclear transcript *VAD* belonging to a class of very long intergenic non-coding RNAs (vlincRNAs) using transfected siRNAs has demonstrated importance of this lncRNA for the maintenance of cellular senescence [107]. RNAi-mediated knockdown of a cytosolic lncRNA *SPRY4-IT1* has showed the role of this transcript in modulation of apoptosis [108]. Inhibition of a spliced lncRNA *ARLNC1* localized in the nucleus and cytosol using either RNAi or ASO technologies has revealed its roles in androgen receptor signaling and growth of prostate cancer cells [109]. Knockdown of a spliced *DGCR5* lncRNA that also localized in both cellular compartments using a mixture of siRNAs and ASOs has demonstrated involvement of this lncRNA in regulating a number of schizophrenia-related genes [110]. Overall, these techniques provided tremendous amount of support for functionality of various known types of lncRNAs. We estimate, based on the analysis of PubMed records, that applications of RNAi and ASO technologies have demonstrated functionality of lncRNAs in > 1500 reports, with RNAi used in the vast majority of those studies. Annotation and functional characterization status of a particular lncRNA could be obtained by querying manually curated databases such as Lnc2Cancer [111], LncRNADisease [112], or LNCipedia [113]. An important note to RNAi- and ASO-based lncRNA functional studies is that most of them have been performed in cultured cells. Nonetheless, the vastness of the lncRNA universe for which the knockdown-associated phenotypes have been shown even led to discussions of potential clinical applications of lncRNA targeting based on these techniques [114].

### Genome targeting

Evidence of lncRNA functionality has also come from other experimental strategies, most notably those that create a complete knockout by altering the DNA sequence of an lncRNA locus [115, 116] (Fig. 1). Application of the CRISPR/Cas9 genome-editing technology has provided support for biological functions of a number of lncRNAs, also predominantly in various cell line models at least in mammalian systems. Genome-editing approaches typically rely on deleting the whole lncRNA sequences or their regulatory regions, since subtle sequence changes require detailed knowledge of functional motifs and domains absent for most lncRNAs. In fact, successful targeted deletions have been achieved over a wide range of DNA sizes. For example, CRISPR/Cas9-mediated hemizygous deletion of a relatively small (~ 700 nt) lncRNA *SPRIGHTLY* (also known as *SPRY4-IT1*) resulted in a decrease in anchorage-independent proliferation rate of cancer cells and the rate of tumor growth in a xenograft model [117]. On the other end of the spectrum, deletion of a 1.1-Mbp region on the human chromosome 6 containing a cluster of vlincRNAs in a fibrosarcoma cell line also using CRISPR/Cas9 has implicated one of them, *vlinc273* or *ASAR6-141*, in control of replication timing of that chromosome [118]. In fact, lncRNA knockouts using genome-editing techniques in cultured cell models implicated lncRNAs in metabolism control [119, 120], cell growth [119, 121–123], metastasis [124], and migration and invasion of human cancer cells [119, 122, 123, 125].

Furthermore, genome editing has also demonstrated functionality of lncRNAs in whole-animal in vivo studies in different animal models (Table 1). In mice, for example, knockout of the *Charme* lncRNA by CRISPR/Cas9-mediated insertion of a polyA cassette into one of its exons resulted in homozygous mice with a specific heart remodeling phenotype (changes in size, structure, and shape of the organ) and reduced lifespan [71]. In nematode and fruit fly, systematic knockouts of multiple lncRNAs resulted in a significant fraction of the mutant animals exhibiting obvious phenotypes. Knockouts of 33 out of 105 testis-specific lncRNAs in fruit fly exhibited a partial or complete loss of male fertility [126]. Importantly, a number of the knockout phenotypes could be rescued by expression of the targeted lncRNAs, strongly arguing that loss of function of the corresponding transcripts caused the observed defects [126]. In *Caenorhabditis elegans*, knocking out 155 out of 170 annotated long intergenic RNAs (lincRNAs) could associate 23 of those with at least one of the 6 analyzed traits [127]. Just like in the previous example, the phenotypes could be either fully or partially rescued by ectopic expression of respectively 9 and 7 of the targeted transcripts [127]. More recently, knockouts of 10 out of 143 multi-exonic lncRNAs via CRISPR/Cas9-mediated deletions in the same species resulted in fertility or growth rate defects in 7 out of the 10 mutants [128]. Furthermore, loss of transcript as the cause of the phenotypes was shown by independent RNAi-mediated knockdowns for 2 out of the 6 tested loci [128].

Recent strategies based on targeting of transcriptional silencers or activators to specific promoters using the CRISPR/dead (d)Cas9 strategy (CRISPR interference (CRISPRi) or activation (CRISPRa)) have also contributed to phenotypic analyses of lncRNAs [129–132] (Fig. 1). For example, CRISPRi-mediated lncRNA knockdown revealed that a radial glia-specific lncRNA *LOC646329* can regulate proliferation in human glioblastoma cells [129]. CRISPRa-mediated upregulation of 4 lncRNAs potentially involved in early cortical cell fate specifications confirmed their roles in regulating genes involved in this process [130].

### High-throughput screening

**Fig. 2:**
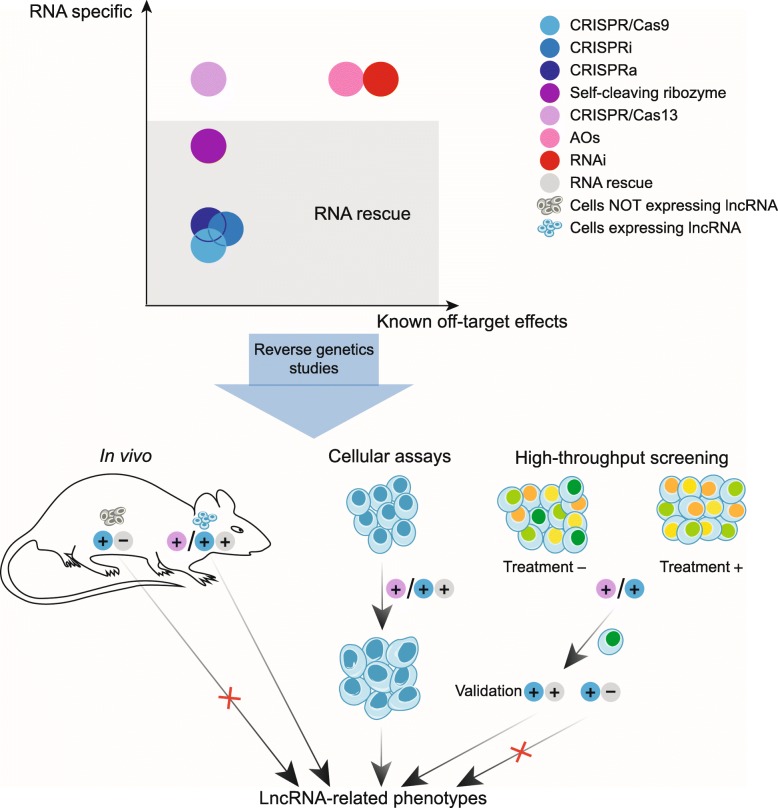
Emerging strategies for investigating biological functions of lncRNAs. Reverse-genetics methods differ as to their abilities to target transcripts and cause off-target/non-specific effects. As such, unambiguous phenotype-lncRNA assignment, especially using methods that do not exclusively target RNA, requires RNA rescue experiments and combination of multiple approaches. Considering the highly specialized patterns of expression for most lncRNAs, in vivo phenotypes are expected to occur only in the cell types expressing the targeted transcript. In contrast, abnormalities happening in the cells that do not express the lncRNA likely indicate transcript-independent effects. On the other hand, cell-based assays have a number of attractive features and remain the only option for lncRNAs whose in vivo expression is not known or with no known homologs in animal models. In cultured cell systems, a phenotypic analysis can be performed either for a single lncRNA (middle) or in a large-scale high-throughput screen (right). More details are in the text

The abovementioned approaches can also be scaled to a whole-genome level analysis in a population-like setting (Fig. 2). In such scenarios, each cell gets tagged or barcoded by an shRNA or a guide (g) RNA sequence targeting a specific transcript and stably integrated into the genome of the cell. Cells harboring tags against transcripts essential for survival would have a tendency to get lost from the population and this loss can be measured by high-throughput sequencing of the barcodes. Such global screens based on the shRNA, CRISPR/Cas9, and CRISPR/dCas9 approaches have also been applied to lncRNA functional studies [133–137].

Importantly, each such survey could identify functional lncRNAs, even though these studies differed in terms of the numbers of targeted transcripts and apparent fraction of phenotypically relevant lncRNAs, thus resulting in very different numbers of the reported functional lncRNAs. For example, using stringent selection criteria, a study with shRNA library targeting 3842 lncRNAs and ultraconserved genomic elements identified one lncRNA *Ntep* as an essential regulator of cell proliferation in NIH3T3 fibroblasts [133]. A dual coding and non-coding integrated CRISPRa screen using 70,290 single guide (sg) RNAs targeting all human RefSeq coding isoforms and 88,444 sgRNAs targeting 14,701 lncRNA genes found the *GAS6-AS2* lncRNA that acts in a *cis*- and *trans*-manner to regulate GAS6/AXL signaling [134]. On the other hand, an shRNA library screen targeting 1280 lincRNAs in the mouse genome identified 20, or 1.6% of these lincRNAs, involved in pluripotency maintenance [135]. Furthermore, a CRISPR/Cas9-mediated genome-scale deletion study of 700 human lncRNAs identified 51 (7.3%) of them as positive or negative regulators of human cancer cell growth [136]. A CRISPRi-based screen targeting 16,401 lncRNA loci in 6 human transformed cell lines and 1 induced pluripotent stem cell (iPSC) identified 499 or 3% of the lncRNA loci required for robust cellular growth [137]. Interestingly, this study also provided a potential reason for the different efficiencies of such surveys: 89% of the functionally relevant lncRNAs showed growth modifying function exclusively in one cell type, indicating a cell-type-specific mode of functioning for many lncRNAs [137]. This finding underscores the need for properly choosing biological systems for lncRNA reverse-genetics studies (also see below). Nonetheless, every reported whole-genome survey could identify at least one lncRNA functionally relevant for the biological system employed.

### Evolutionary conservation

Contribution of a genomic element to survival of a species or species over multiple generations in real-life field conditions arguably represents the ultimate test of functional significance of that genomic feature. While such experiments are very challenging to conduct in vivo for practical reasons (also see below), the availability of genome sequences for a multitude of species allows to estimate the ability of a genomic element to withstand natural processes of sequence change or loss during evolution. In other words, the genome of any given species (or individual) contains information on a myriad of past survival experiments conducted over millions of years of evolution and comparison of multiple genomes among or within multiple species allows to extract it. Any genomic sequence that changes less than expected from random chance (or, in other words, remains conserved) across genomes of multiple species is widely assumed to represent a genomic feature that contributes to survival even if the function of the latter is unknown. And, indeed, such assumption has been exhaustively validated on exons of protein-coding genes where the general trend of primary sequences conservation across multiple species is very obvious.

In contrast, with notable exceptions of some highly conserved lncRNAs such as *Neat1* and *Malat1* [46], in general, primary sequences of mammalian lncRNAs do not exhibit features consistent with evolutionary conservation [138]. Such studies have some important caveats, however, as reviewed by Pang et al. [139]. Of special note, lncRNA evolutionary conservation signatures can become apparent when features other than primary sequences are compared, specifically RNA 2D structures [140], transcript exon-intron structures [141], relative positions in the genomes (synteny), and expression patterns [142]. Specifically, conservation of an exon-intron structure implies selective pressure on the transcript rather than DNA sequence elements that might overlap it [141]. Strikingly, using this approach, Nitsche et al. revealed conservation of over 70% of 5413 human lncRNAs across major eutherian families [141]. Furthermore, Hezroni et al. estimated that over 1000 human lincRNAs have conserved functions in mammals based on conservation of synteny and expression patterns across 17 species [142].

## lncRNAs appear to be dispensable for a vertebrate organism

Despite the ample body of evidence supporting general biological relevance of the lncRNA class of transcripts reviewed above, derived primarily from studies on cultured cells, multiple in vivo reverse-genetics probes into their function done in vertebrate organisms challenge this conclusion. The first indication that the non-coding genome is dispensable for their survival came in 2004 when Nobrega et al. reported on strains of mice engineered to have deletions of two large non-coding gene desert regions, 1511 kb and 845 kb in lengths, harboring 1243 non-coding sequences conserved between humans and rodents [143]. Mice homozygous for the deletions had no distinguishable changes in multiple general homeostasis criteria [143]. While the presence of lncRNAs in the deleted regions was not assessed, considering that based on the ENCODE consortium’s estimates, up to 75% of the human genome is transcribed [3], it is highly likely the large deleted regions do in fact encode such transcripts.

Recently, Han et al. knocked out 12 lncRNAs from the mouse genome, including 9 lncRNAs conserved syntenically in the human genome, 8 located near developmentally important genes, and 4 previously reported to play important biological roles based on studies in cultured cells [21]. Despite the fact that the selection of the targets was supposed to enrich for developmentally important lncRNAs, the authors found that mice homozygous for knockouts of 11 out of the 12 lncRNAs were born at the expected Mendelian ratios and were viable with no obvious abnormalities [21]. The phenotype of the remaining lncRNA knockout was attributed to the deletion of a DNA sequence, rather than the transcript itself (see below) [21]. In 2019, Goudarzi et al. used the CRISPR/Cas9 approach to knockout 25 zebrafish lncRNAs [22]. Just like in the study above, the authors have carefully selected these transcripts based on conservation, expression patterns, and proximity to genes important in development to maximize the possibility of biological relevance of these lncRNAs [22]. Strikingly, although some might affect transcription of neighboring genes, none of the 25 lncRNAs were required for embryogenesis, viability, or fertility [22].

In addition to the failure of generating in vivo phenotypes, reproducing those that could be obtained appears less than certain (Table 1). Perhaps the most striking example of this concerns one of the most studied lncRNAs, *Hotair*, whose biological significance recently became a subject of debate [26, 50, 144, 145]. In 2013, Howard Chang’s lab reported that a targeted homozygous deletion of *Hotair* in the mouse genome led to homeotic transformation, derepression of genes including *HoxDs*, and skeletal malformations [50]. However, in 2016, Denis Duboule’s lab obtained the *Hotair* deletion strain of mice from the Chang’s team and crossed it with animals of a different background [26]. They found no detectable change in the *HoxD* gene expression and no significant morphological alterations in the progeny harboring the homozygous deletion of the lncRNA [26]. Overall, of the 3 anatomical phenotypes associated with the *Hotair* knockout reported by the Chang’s group, 2 could not be found by the Duboule’s team at all and one was found in a very subtle form and attributed to DNA-dependent events [26]. Furthermore, the Duboule’s team could not reproduce any of the previously reported effects of the *Hotair* knockout on gene expression [26]. In another example, the *Gomafu* lncRNA was associated with an anxiety-like behavior in mice where this transcript was knocked down in the medial prefrontal cortex using ASOs [146]. However, a later study in knockout mice lacking *Gomafu* in the entire brain showed no difference in the same behavioral tests [79].

To further compound the problem with in vivo phenotypes, those initially believed to associate with lncRNAs can actually be caused by different mechanisms. For example, the *Linc-p21* lncRNA has been extensively studied due to its involvement in p53 signaling, human diseases and has been reported to regulate various biological processes [28, 147–149]. It may function in *cis* [28] or *trans* [147]. However, an in vivo study using a mouse knockout model showed that deletion of the locus significantly affected local gene expression even in tissues with no detectable *Linc-p21* expression, suggesting that DNA enhancer elements in the *Linc-p21* locus rather than the transcript itself are responsible for this effect [150]. In another example, mice engineered to have insertion of a polyA cassette into the *Hand2as* lncRNA showed right ventricular hypoplasia and embryonic lethality phenotypes, thus associating this transcript with heart development [101]. However, a later study that created separate deletions of the *Hand2as* gene body and promoter regions with the CRISPR/Cas9 technology found only the former to have heart-related defects, thus arguing that the phenotypes were caused by the DNA locus rather than the lncRNA [21].

Even for the lncRNAs generally considered as the “gold standards,” the situation with the strength of evidence for their in vivo functionality is not straightforward (Table 1). *Xist*, *H19*, *roX*, *Neat1*, *Malat1*, and *Hotair* are perhaps the most well-studied lncRNAs accounting for at least 4500 records in PubMed. For example, in the case of *Malat1*, a number of studies using cultured cells associated this lncRNA with the regulation of gene expression [151] and a variety of biological processes like pre-mRNA splicing, cancer cell metastasis, cell cycle progression, and serum-induced cell proliferation [152–155]. However, in 2012, Eissmann et al. [23], Nakagawa et al. [25], and Zhang et al. [46] independently generated *Malat1* knockout mice and found the homozygous knockouts to be viable and fertile, with no obvious phenotypes or histological abnormalities, including no obvious defects in nuclear speckles where this lncRNA localizes. Furthermore, despite significant evidence of involvement of *Malat1* in hypoxia response and specifically in renal ischemia-reperfusion injury, no discernable in vivo effect of the lncRNA on that condition was observed in a mouse knockout [24]. On the other hand, in vivo effect of *Malat1* in brain tissues after ischemic stroke could be observed; however, no RNA rescue experiments have been conducted in those studies [48] (also see below for additional discussion of *Malat1* in vivo studies). In summary, strikingly, even among those transcripts, clear and uncontroversial evidence of biological function in vivo exists only for very few (Table 1). In fact, among the “gold standard” lncRNAs mentioned above, consistent in vivo phenotypes that could be restored in RNA rescue experiments thus unequivocally attributing the phenotypes to the corresponding transcripts were only reported for *roX* and *H19* (Table 1).

All in all, the points discussed above clearly show that a very large gap exists between the abundance of data demonstrating biological function of lncRNAs in cultured cells and the difficulty in obtaining such evidence in in vivo studies. Below, we will try to provide reasons, both technical and biological, that might explain this discrepancy and try to reconcile potential biological functionality of the lncRNA class of transcripts with these observations.

## Targeted lncRNA is not always the cause of the phenotype attributed to it

### Non-specific or off-target effects in reverse-genetics assays

The most trivial explanation for the discrepancies described above is that phenotypes observed after lncRNA knockdowns or knockouts are not related to these transcripts. Indeed, a number of recent reports suggest that this is a likely possibility. As mentioned above, RNA depletion strategies using RNAi or ASOs account for majority of the phenotypic evidence. Most often, such studies are done by transfecting synthetic siRNA or ASO molecules into cultured cells. However, this can lead to supraphysiological amounts of the synthetic molecules inside the cells leading to formation of aberrant RNA species that can cause non-specific changes in gene expression [156] and potentially cause phenotypic changes unrelated to the intended targets. Furthermore, both RNAi and ASOs have well-recognized non-specific and off-target effects [157–165] that are very hard or even impossible to completely avoid and non-trivial to control for [162, 166]. For example, a recent report by Stojic et al. found that transfection of non-specific siRNAs or ASOs can cause substantial transcriptome changes in a sequence-specific manner [162]. This observation has huge practical implications since vast majority of siRNA/ASO studies use a single control of unrelated or scrambled sequence to estimate non-specific effects of the targeting siRNAs/ASOs.

These results imply that RNAi- and ASO-based studies potentially have non-specific effects unaccounted for and, logically, these effects could in fact be responsible for the observed phenotypes. Indeed, this has been shown to be the case. Goudarzi et al. injected morpholino antisense oligonucleotides (MOs) against the lncRNA *cyrano* into homozygous zebrafish deletion mutant lines with no corresponding target sequences for that lncRNA present [22]. Strikingly, they could reproduce all phenotypes previously reported based on injection of the same MOs into wild-type animals, suggesting that the phenotypes were caused by non-specific effects of the MOs rather than by the knockdown of the lncRNA [22]. Furthermore, Kok et al. generated a zebrafish mutant with a segment of the lncRNA *megamind* targeted by previously published MOs deleted [167]. Injection of the same *megamind* targeting MO into this mutant led to the same biological effects as in the wild-type, again strongly suggesting the off-target effect of the MOs as the root cause of the phenotypes originally attributed to the *megamind* knockdown [167]. The problem with phenotypes caused by non-specific effects is not limited to lncRNAs. For example, RNAi-mediated phenotypes initially associated with knockdown of the protein-coding fruit fly gene *pico* could not be rescued by an RNAi-resistant *pico* sequence, again suggesting that the gene was not connected to the observed phenotypes [163].

Furthermore, it is not clear which knockdown method is more specific. Unfortunately, non-specific effects are not limited to siRNAs; shRNAs have also been shown to have them, partially through interfering with the immune response and miRNA levels in the cell [164]. By comparing transcriptome profiles of knockdowns of the same transcripts using siRNAs, ASOs, and CRISPRi, Stojic et al. found very little overlap among the genes whose expression changed in response to the knockdowns, suggesting the existence of method-specific off-target effects [162]. While the authors suggested that CRISPRi had the fewest numbers of the off-target effects, consistent with the currently prevalent notion that the CRISPR/Cas9 technology in general has high precision and fidelity [168], growing evidence suggests that the off-target effects in this system are also non-negligible [169–171]. For example, among 12 tested sgRNAs, the off-target binding sites of dCas9 ranged from ~ 10 to > 1,000 in the human genome [169]. Two more recent studies found that Cas9-mediated cytosine base editor has substantial off-target effects in the rice and mouse genomes [170, 171].

Theoretically, targeting multiple sites within the same transcript should increase the reliability of assigning the phenotypes to the transcript. Multiple studies using siRNAs or ASOs rely on this strategy to account for the off-target effects, with as many as 5 independent ASOs per transcript [172]. Presumably, non-specific effects of different siRNAs or other molecules targeting the same transcript would be different, while the common phenotypes should represent the true effect of the targeted transcript. However, while two or three independent MOs targeting respectively the *cyrano* or *megamind* lncRNAs produced similar phenotypes [173], these MOs were later shown to cause the phenotypes by effects other than the knockdowns of the target lncRNAs [22, 167]. Still, although phenotype-transcript associations obtained using such strategy do not necessarily represent the underlying biological truths, the strategy represents probably the most essential control for the off-target effects in siRNA/ASO-based experiments and as such must always be followed.

### Transcript-independent causes of phenotypes

Multiple studies are pointing to the fact that an lncRNA locus may not necessarily function only via the transcript itself. For example, using genome-editing techniques, Engreitz et al. found that of the 5 lncRNA loci that can influence expression of the neighboring genes, none in fact required the transcripts to mediate this effect [174]. Instead, the phenomenon was mediated by a regulatory cross-talk between neighboring genes also known to occur between protein-coding loci [174]. The lncRNA *Airn*, located in a well-characterized imprinted locus, is antisense to *Igf2r* and was believed to function by silencing this protein-coding gene [98, 175]. However, a study employing a series of shortened endogenous *Airn* lncRNAs showed that the overlap of the lncRNA transcription with the *Igf2r* promoter was responsible for the silencing and excluded a role of the lncRNA in this phenomenon [102]. These findings explain quite well the abovementioned cases of the lncRNAs whose original biological functions were later reclassified as not attributable to the transcripts themselves. Unfortunately, reverse-genetics strategies that do not exclusively target a transcript may incorrectly associate it with a biological process. All in all, while multiple in vivo phenotypes for mouse lncRNA knockouts have been reported, only a handful of those were confirmed by RNA rescue experiments (Table 1), thus leaving a possibility open that the observed defects were not caused by the targeted transcripts per se (Table 1).

## Can lncRNAs still have biological functions?

While it is hard to estimate the fraction of lncRNAs whose reported phenotypes are affected by the issues described above, the consistent emergence of reports pointing to the problems with functional studies of lncRNAs suggest that this fraction might be significant. Moreover, the recent studies point to the fact that a true in vivo phenotype (i.e., truly caused by the transcript per se) of any given lncRNA knockout at least in vertebrates would likely be subtle if at all observable. This brings a natural question of whether these transcripts can be functional at all and, if so, how these functions can be reconciled with the abovementioned phenotypic studies. Below, we will review studies that potentially point to possible modes of biological functionality of lncRNAs that could in turn explain the controversial results described above.

### Subtle effects

A hint to a mode of lncRNA functionality could potentially come from the genome-wide association studies (GWAS). The meta-analysis of these studies shows that most of the phenotype-associated polymorphisms lie in the non-coding parts of the genome [176] and their effects are rather small [177]. While the polymorphisms can function via altering DNA regulatory sequence elements [178], it is quite conceivable that they might function by affecting lncRNAs as well [179]. In fact, the greatest known risk factor for atherosclerosis mapped by GWAS to 9p21.3 was attributed to the lncRNA *ANRIL*, believed to function by regulating multiple genes in *trans* [180, 181]. Single nucleotide polymorphisms in the antisense lncRNA *RP11-634B7.4* have been associated with severity of pre-treatment pain in head and neck cancer patients [182]. Furthermore, through extensive analysis of expression profiles of human lncRNAs, the FANTOM consortium found that 1970 lncRNA genes associate with at least one GWAS trait [183].

In such scenario, the small effect sizes typically observed in GWAS studies would be consistent with the subtle phenotypes of lncRNA knockouts. In this model, each lncRNA would contribute a small effect, yet due to their vast numbers, resulting in a significant cumulative biological impact of these transcripts [179].

### Cell-type-specific functions

lncRNAs are well known to have highly cell-type-specific patterns of expression, much more so than protein-coding mRNAs. As shown by the ENCODE consortium, only 10% of lncRNAs were constitutively expressed as compared to 53% of mRNAs based on expression analysis across multiple human cell lines [3]. On the other hand, 29% of the former were detected only in one cell line compared to 7% of the latter [3]. Highly cell-type- and temporal-specific lncRNA expression patterns have also been shown in vivo [184–187]. Analyses of patterns of expression of various lncRNAs in mammalian brains based either on in situ hybridization [184] or RNA-seq analysis [185] revealed highly restricted patterns confined to neuroanatomical regions, cell types, or subcellular compartments in a gender-dependent fashion. And, consistent with this theme, lncRNAs tend to have narrower time windows of expression than mRNAs during early development [186].

The restricted expression feature of lncRNAs fits well with the abovementioned results of the CRISPRi phenotypic screen where 89% of the positive lncRNAs displayed the phenotypes exclusively in one cell type [137]. Obviously, this feature would significantly complicate detection of a phenotype in vivo since without prior knowledge of the expression patterns of a target lncRNA, the phenotype could be easily missed. Unfortunately, since many lncRNAs were found and characterized in cultured and (predominantly) cancerous cell lines, their in vivo expression profiles are not known.

### Functional redundancy

Functional redundancy of genes is a strategy formed during evolution to counter adverse effects of mutations in genes encoding critical molecular components [188, 189]. And, because of this, knockout of a single gene or its functional element may not show a phenotype [190, 191]. For example, genes encoding some of the main cell cycle regulators such as *Cdk2*, *Cdk4*, and others were found to be non-essential for survival in vivo [192, 193]. However, double knockout of *Cdk2* and *Cdk4* caused embryonic lethality, demonstrating that *Cdk2* and *Cdk4* function redundantly to couple the G1/S phase transition to mitosis [194]. Similarly, the mice lacking either of the sorting nexin family genes *Snx1* and *Snx2* are viable and fertile, while the double mutant is embryonic lethal, indicating that these genes have essential yet redundant functions [195]. The PINCH proteins are the key components of the integrin signaling pathway. The mice with cardiac-specific ablation of PINCH1 or germline ablation of PINCH2 displayed no basal cardiac phenotype [196, 197], while the mice with cardiomyocyte-specific double knockout of these genes showed cardiomyopathy, heart failure, and early postnatal lethality [198].

This is also true in the lncRNA world—neither one of the *Drosophila roX1* or *roX2* genes is essential for survival, while the double mutant is male lethal [39]. Somewhat similar situation has been also observed with *Malat1* (Table 1). As mentioned above, the three independently generated *Malat1* knockout strains of mice showed no obvious phenotypes [23, 25, 46]. However, crossing these *Malat1* knockouts into genetic backgrounds of breast cancer and atherosclerosis mouse models could in fact reveal in vivo effects of the lncRNA on these ailments [45, 47, 49], although the two breast cancer studies showed contradictory results in terms of the direction of the effect [45, 47] (Table 1).

As illustrated by these examples, obtaining an obvious or observable phenotype sometimes requires knockout of several genes. However, this would present a significant complication in an lncRNA functional study not only because of the technical challenges caused by targeted knockouts of multiple lncRNAs in the same genome, but also because the redundant elements for an lncRNA are typically unknown. It is thus reasonable to suggest that at least in some phenotypic studies, the true functions of lncRNAs were masked by other functionally redundant genes.

### Missed phenotype

The phenotype of a given lncRNA can be outside of the scope of the tests performed on the knockout animals. For example, *Neat1* lncRNA exclusively localizes to paraspeckles and serves as an architectural component of these nuclear bodies as shown by reverse-genetics studies in cultured cells [106, 199]. Knockdown of this lncRNA in cultured cells caused disruption of the paraspeckle structure [106, 199], while overexpression of *Neat1* led to an increase in paraspeckle numbers [106]. On the other hand, Nakagawa et al. reported that *Neat1* homozygous knockout mice lacked paraspeckles, yet were viable and fertile, indicating that these nuclear substructures are not essential in vivo and leaving the biological function of *Neat1* unresolved [52]. Later, the same group discovered that naturally mated female knockout mice had impaired ability to get pregnant due to defects in formation of *corpus luteum*, where *Neat1* is expressed in adult animals (also see below) [53]. In the same year, another group also found *Neat1* in paraspeckles of the mammary gland luminal epithelial cells and essential for mice mammary gland development and lactation [54]. Later on, *Neat1* was also found to have in vivo effects under some other specific conditions inducing expression of this lncRNA [55–57]. For example, *Neat1* can be induced by activation of *p53* and ablation of this lncRNA can lead to impaired tumorigenesis in mice [55].

Special consideration has to be given to a possibility that a mutant phenotype may become apparent only in natural conditions as revealed by behavioral analyses of BC1 knockout mice [200]. The mutant animals lacking this small (~ 150 nt) non-coding RNA expressed in neurons have no obvious anatomical or neurological defects [201]. However, the mutant mice had decreased exploration behavior under outdoor semi-naturalistic settings, leading to failure to locate distant food sources and higher mortality compared to the wild-type animals [200]. The phenotype was consistent with evolutionary conservation of the BC1 sequence among rodent species [200], but it would not be revealed under standard laboratory conditions. Noteworthy, the abovementioned study by Akay et al. also failed to detect obvious phenotypes in the 10 *C. elegans* lncRNA knockouts [128]. Only extensive analysis of the mutants alongside the wild-type animals using automated microscopy could reveal the phenotypes affecting individual and population fitness [128].

In summary, even in the protein-coding gene realm, it is common for a knockout animal to have either no observable phenotype or a phenotype revealed only under certain environmental or genetic conditions [202]. Although the authors in the above examples were fortunate in finding the in vivo phenotypes for *Neat1* and BC1 (albeit without RNA-based rescue confirmation) and other lncRNAs either in specific cell types or under specific environmental conditions, it is quite possible that some viable and fertile lncRNA knockout animals may harbor yet undiscovered issues associated with the absence of these transcripts.

## Emerging solutions to address the challenge of uncovering true biological relevance of lncRNAs

### Unequivocal assignment of phenotypes to lncRNA transcripts

Based on the examples described above, it would perhaps not be an exaggeration to state that for many if not most lncRNAs the authenticity of the reported phenotype-transcript associations in any system is still ambiguous. It is fairly clear that the main reason for it lies in the issues with the currently used reverse-genetics methods and strategies described above (Fig. 1, also reviewed in Cao et al. [8]). Thus, new technologies and experimental strategies are badly needed. The progress in this area is occurring in at least three directions.

First, development of new RNA-targeting knockdown methods with significantly reduced non-specific and off-target effects. One such promising approach is represented by the newly developed CRISPR/Cas13 system from *Leptotrichia wadei* (LwaCas13a) that can be programmed to target a specific transcript via agRNA specifically designed against the latter [203] (Fig. 1). This system could reportedly knockdown nuclear localized lncRNAs such as *MALAT1* and *XIST* [203]. Furthermore, the CRISPR/Cas13 method was reported to have comparable knockdown efficiency as RNAi, but with substantially reduced off-target effects [203]. Finally, 2 mutations in the middle of a 28-nucleotide gRNA (representing only 7% of the sequence) greatly reduced the efficiency of knockdown, thus allowing for a perfect mutant non-targeting control for each gRNA [203]. This feature potentially gives the CRISPR/Cas13 system an additional strong advantage over methods like RNAi or ASOs where such small sequence changes would not likely abrogate the targeting effects [166] and thus cannot be used to design matching controls.

Our group applied the CRISPR/Cas13 technology to investigate the functionality of vlinc class of nuclear lncRNAs in a large-scale setting [204]. For each vlincRNA, we designed 10 targeting gRNAs and 10 non-targeting mutant control gRNAs differing from the former by 3 mutations in the center of the 28-mer gRNA [204]. We have generated a population of human cells constitutively expressing targeting and non-targeting control RNAs in the background of inducible Cas13 [204]. We then assessed changes in the abundance of targeting gRNAs relative to the non-targeting controls for each vlincRNA in response to Cas13 induction and in the context of treatments with various anticancer drugs previously found to upregulate these vlincRNAs at the expression level [204]. Overall, we could find that 64% (16 out of 25) of the tested vlincRNAs were relevant for cellular survival in response to the anticancer drug treatments [204].

Another promising new approach is insertion of self-cleaving ribozymes into lncRNA sequences (Fig. 1). In fact, this strategy has been applied in lncRNA functional studies and resulted in 50–90% inhibition of the target transcripts, comparable to the other knockdown approaches [205]. This interesting approach has a number of attractive features: (1) depletion should be limited only to the transcript harboring the ribozyme sequence and, as such, should not have any off-target or non-specific effects; (2) it should have minimal effect on the genomic locus (compared to a deletion) and theoretically should not interfere with the process of transcription; (3) it can also work in the nucleus; (4) the ribozyme can be inactivated by point mutations, thus creating a perfect control for any non-specific and off-target effects; and (5) it can be reversed by blocking ASOs or chemical inhibitors to allow for the rescue experiments. However, the method also has some limitations: (1) it involves CRISPR/Cas9-mediated targeted insertion of a ribozyme sequence, making it more complex than other RNA-targeting knockdown methods, and (2) it has the potential to disrupt a functional DNA element overlapping an lncRNA.

Second, the realization that a combination of multiple approaches and/or mutant alleles is needed to fully understand the root cause of a phenotype (Fig. 2). For example, as illustrated in the *Hand2as* case above, a phenotype truly associated with an lncRNA should be found in deletions of both the gene body and promoter, while the lack of concordance likely indicates transcript-independent functions [21, 101]. In another example, the authors used a combination of different methods to separate functions of DNA sequence elements, transcription, and the transcript within the same lncRNA locus [206]. They first performed CRISPR/Cas9-mediated knockout and ASO-mediated knockdown of the *BGLT3* lncRNA and found a reduction in transcription of the *γ-globin* genes that could be rescued by overexpressing *BGLT3* in the knockout cells [206]. The authors also employed CRISPRi to suppress the *BGLT3* transcription and found a reduction of the *γ-globin* transcription that could not by rescued by overexpressing *BGLT3* in these cells [206]. Taken together, the authors provided clear integrative evidence that both the *BGLT3* transcription and transcript can upregulate transcription of the *γ-globin* genes [206]. A DNA locus and the corresponding transcript can sometimes have distinct or even opposing roles. In embryonic stem cells, CRISPR/Cas9-mediated genomic deletion of the lncRNA *Haunt* downregulated the *HOXA* gene cluster, while depletion of the *Haunt* transcript by RNAi, polyA insertion, or promoter deletion upregulated the *HOXA* genes [207]. However, restoring expression of the* Haunt* transcript via knock-in into the original genomic location in the background of the homozygous *Haunt* deletion mutant could not rescue the downregulation of the *HOXA* expression [207]. The authors concluded that the *Haunt* genomic locus contains DNA elements with potential enhancer functions for the *HOXA* genes while the lncRNA can potentially silence them [207].

In summary, a single reverse-genetics method is unlikely to yield a conclusive answer as to lncRNA biological function especially if it does not explicitly target RNA, calling for a combination of multiple approaches and careful analysis to separate different possible causes of the observed effects. However, caution should also be exercised when interpreting inconsistent results from different experimental approaches because each reverse-genetics method might target unique pools of transcripts derived from the same locus. For example, while both ASO- and RNAi-mediated knockdowns resulted in similar levels of depletion of the lncRNA *linc-HOXA1*, only the former method affected a specific subset of *linc-HOXA1* RNA molecules—those associated with sites of transcription—and led to the *cis* phenotype of suppression of the nearby *Hoxa1* gene expression [208].

Still, presence of several DNA elements with different functions overlapping an lncRNA transcript—not an unlikely scenario—could theoretically mislead interpretation of even carefully constructed genome-editing experiments (Fig. 1). As such, there is a growing realization that RNA-based rescue experiments are required in lncRNA functional studies based on genome-editing tools (Fig. 2). For example, in addition to the studies in nematode and fruit fly described above [126, 127], a handful of mammalian knockout phenotypes have been validated by RNA rescue (Table 1). For example, restoring the *Fendrr* lncRNA expression in the corresponding knockout rescued the majority of the abnormal phenotypes in the heart and body wall development [58]. Likewise, ectopic expression of the *Firre* lncRNA in the *Firre* knockout mouse rescued the defects in hematopoiesis and alterations in gene expression [59]. More interestingly, through RNA-based rescue, phenotypes of the *H19* knockout mice previously attributed to the *cis* effect of the H19 locus on the local chromatin environment [32, 33] were proven to be also caused by the *H19* transcript itself [34] (Table 1). However, such experiments could be quite challenging for lncRNAs. First, the size of many lncRNAs, particularly the vlincRNA species with the lengths over 50 kb [209], makes their overexpression technically difficult. Second, the nature of the functional transcript may not always be known. For example, some lncRNAs like *Xist* and *Neat1* have multiple isoforms, which may possess different functions [52, 210]. Third, ectopic expression would not work in cases where the genomic locations are important, for example in the cases of *cis*-regulatory lncRNAs which could be numerous in the human genome [9]. In fact, as mentioned above, such validation is rare even for the “gold standard” lncRNAs. Finally, it is noteworthy that even positive RNA-based rescue outcomes may sometimes have flaws. For example, the *megamind* and *cyrano* lncRNAs were proven to be functional via RNA-based rescue [173]; however, later studies found that the observed phenotypes were due to non-specific effects of the reverse-genetics method employed [22, 167]. Still, in the contexts of the techniques that cannot exclusively target transcripts, RNA-based rescue experiments would likely remain critical in providing unambiguous connections between lncRNAs and phenotypes.

### Choice of a biological system for reverse-genetics studies

A phenotype un-ambiguously attributable to an lncRNA in an animal model would always hold the key to answering the question of whether the lncRNA has biological function. However, obtaining such a phenotype is extremely challenging, not in a small part due to a highly restricted expression pattern of a typical lncRNA that makes obvious, global defects caused by its knockout less likely. Indeed, the phenotype(s) would likely associate with the cells or tissues expressing the lncRNA (Fig. 2), as exemplified by *Neat1* where the phenotypes were found in specific cell types and conditions expressing this transcript (see above). In another example, expression of the *Firre* lncRNA is the highest in the hematopoietic stem cells and, as expected, knockout of that lncRNA in mice caused defects in the hematopoiesis [59]. Still, a knockout of an lncRNA expressed in a limited number of cells in an adult animal or even fetus is less likely to cause an obvious defect. As such, embarking on an in vivo phenotypic experiment would only be warranted if the expression profile of a target lncRNA is reasonably well understood. In this respect, while the community has access to a number of comprehensive expression datasets in humans (ENCODE [211], GTEX [212], TCGA [213]), mouse (Mouse ENCODE [214]) or both (FANTOM5 [183, 215]), an atlas of the spatio-temporal expression patterns of lncRNAs in animal models would be highly desirable for any future reverse-genetics studies.

On the other hand, functional studies on cultured cells are unavoidable in a number of scenarios and also offer a number of advantages compared to the whole-animal studies (Fig. 2). First, as mentioned above, many lncRNAs have been found only in cell lines and their patterns of expression in vivo are not known. Second, due to low sequence conservation of mammalian lncRNAs [138], the homologs of human transcripts in animal models may be unknown due to deep divergence in sequence and structure [216] or may not even exist. Third, human lncRNAs found only in cell lines can still have properties making them attractive for in-depth analysis, for example involvement in drug resistance, leaving the cell lines as the only logical choice for these assays. Fourth, cell lines are significantly cheaper and easier to manipulate than animals. Finally, cultured cells allow for the high-throughput population-level assays ideally suited for detecting subtle phenotypes based on measuring small changes in cell populations by deep sequencing of barcodes inserted into the cells. As described above, such strategies based on libraries of shRNAs or sgRNAs in RNAi-, CRISPR/Cas9-, CRISPR/dCas9-, or CRISPR/Cas13-based assays could annotate biological functions of the target genes based on detection of subtle changes in viability or stress resistance as shown in multiple studies [133–137, 204]. Importantly, large-scale screens allow relative quantitation of the effect of each lncRNA on cellular fitness by measuring fold change—depletion or enrichment—for each shRNAs or sgRNAs. This would allow for ranking of all lncRNAs according to their biological effects in multiple cell types—something that would be hard to achieve in in vivo studies. Arguably, such studies using proper controls and multiple targets against each transcript could indeed be quite revealing in annotating biological functions of lncRNAs (Fig. 2).

Finally, it should also be realized that an lncRNA may in fact encode short peptide(s) and thus represent an mRNA. Moreover, such peptides can have biological functions as revealed by the phenotypes of the corresponding knockouts. For example, a spliced human transcript originally annotated as the lncRNA *LINC00948* was later realized to represent an mRNA encoding a 46-amino acid micropeptide myoregulin [217]. In vivo knockout of the peptide resulted in a muscle performance phenotype [217]. The number of such lncRNAs turned mRNAs encoding functional peptides is steadily growing [103, 218, 219]. Since the presence of a peptide-encoding open reading frame might be difficult to discern from sequence analysis alone, it is quite probable that an lncRNA initially associated with a specific biological process might actually function as an mRNA.

## Conclusions

Perhaps the major challenge in the lncRNA field is to prove beyond a reasonable doubt the biological significance of these transcripts not only in cultured cells but also at the organismal level. As of now, in vivo phenotypes in reverse-genetics studies appear rather subtle and/or highly redundant for most of these transcripts. However, the challenges posed by these negative outcomes may also represent opportunities as we might be getting hints as to the actual modes of functioning of lncRNAs in vivo. However, novel experimental methods and strategies have to be adopted to match these challenges and to resolve the debate about the functionality of this fascinating class of RNAs.

## Supplementary information

**Additional file 1.** Review history.
