# Supplementary material for: Reverse-genetics studies of lncRNAs—what we have learnt and paths forward
Source: Genome Biol. 2020 Apr 14;21:93. doi: 10.1186/s13059-020-01994-5 (PMC7155256; doi:10.1186/s13059-020-01994-5)
Supplement: Supplementary file 1 — Additional file 1. Review history. [file 13059_2020_1994_MOESM1_ESM.docx]

**First round**

**Reviewer 1**

**Is the topic of the article timely and of interest to a wide range of readers?**

Yes

**Is the article well written and presented in a logical way?**

Yes

**Do the authors cover the relevant literature in an accurate and balanced way?**

Yes

**Do the authors provide insightful discussion of the future directions for the field?**

Yes. The authors discuss future directions.

**Do any tables, figures or boxes that are present enhance the manuscript?**

Yes

**Would the manuscript benefit from any additional tables, figures or boxes?**

No: I am satisfied with the table and figure

Gao and colleagues present a review of the current state of research into the functions of lncRNAs, and particularly into how the various perturbation tools should be interpreted. The review is certainly timely and addresses a question of major importance. My comments are relatively minor.

- Antisense oligonucleotides are more often referred to as "ASO" not "AO"

- It should be mentioned that siRNAs often give different results to ASOs for the same target, and it has been hypothesised that this may be due to the fact that siRNAs are targeting cytoplasmic populations of the lncRNA, whereas ASOs get the nuclear population. See for example Maamar et al PMID 23723417

- I found the section 'Evidence supporting the biological roles of lncRNAs" a little formulaic, citing various studies one after the other, without enough synthesis and interpretation of the overall results.

- Section "LncRNAs appear to be dispensable for a vertebrate organism". While I do agree with the arguments put forth, the authors in my view do not give enough weight to data arguing against these findings. There are numerous papers on protein coding genes that yield no phenotype, or extremely subtle phenotypes such as FOXP2. There are papers showing phenotypic effects of knockout of ultraconserved elements. There are papers showing that lncRNA phenotypes only become apparent in more natural field trials (https://doi.org/10.1016/j.bbr.2004.02.015). The final criterion of functionality is in the survival fitness of animals in the wild - which is far from being modelled in laboratory conditions. This is the most likely explanation for extremely highly conserved and abundant RNAs like MALAT1 or NEAT1, which yield such subtle phenotypes in knockout animals.

- In general I think the authors pay too little attention to evidence for lncRNA functionality from evolutionary sources. There is clear evidence from Haerty+Ponting, Ulitsky, Stadler (https://www.ncbi.nlm.nih.gov/pmc/articles/PMC4408788/) that lncRNAs are under purifying evolutionary selection. Such selection would be hard to reconcile if lncRNAs did not play roles in fitness.

-Table 1: I found this a very useful resource, since I'm not aware of any table of lncRNA knockout animals. I would encourage the authors to make this more comprehensive to cover all the present mouse knockouts, which I understand only amount to a handful at present.

- Page12: I don't find it helpful to so extensively discuss your own unpublished paper, since the author is unable to read it. This could be remedied by submission to Biorxiv at least.

- Evidence from C elegans: Another recent paper demonstrating functional lncRNAs in worm: <https://www.ncbi.nlm.nih.gov/pubmed/30777050>

**Reviewer 2**

**Is the topic of the article timely and of interest to a wide range of readers?**

Yes

**Is the article well written and presented in a logical way?**

Yes

**Do the authors cover the relevant literature in an accurate and balanced way?**

Yes

**Do the authors provide insightful discussion of the future directions for the field?**

Yes. Gao and colleagues have produced a timely review on lncRNA functionality by bringing together studies at both end of the spectrum. Their manuscript provides a great overview of the potential issues associated with individual genetic approaches aimed at perturbing lncRNA expression or function. They also provide several suggestions on how to approach the problem.**Do any tables, figures or boxes that are present enhance the manuscript?**

Yes

**Would the manuscript benefit from any additional tables, figures or boxes?**

No. The manuscript text is sufficiently clear and doesn't require further support by additional tables or figures.

Gao and colleagues have produced a timely review on lncRNA functionality by bringing together studies at both end of the spectrum. Their manuscript provides a great overview of the potential issues associated with individual genetic approaches aimed at perturbing lncRNA expression or function. They also provide several suggestions on how to approach the problem. I only have a couple of minor comments that are worthwhile considering.

The lncRNA database lncipedia contains > 1000 papers describing functions of individual lncRNAs. They authors may also refer to this when discussing literature support for lncRNA functionality.

On page 7, when discussing off-target effects of siRNAs/AOs, the authors may which to add that most studies use multiple siRNAs/AOs against the same target lncRNA to demonstrate on-target effects and cancel out off-target effect. They could also make suggestions to the readership that, when working with these strategies, using multiple siRNA/AOs is a must.

On page 14, the authors state that many lncRNAs have been found in cell lines and in vivo expression patterns are not known. While we are clearly missing information on the spatio-temporal expression, we do have access to in vivo expression datasets like the ones generated by TCGA, GTEX, FANTOM, … that do provide in vivo expression of lncRNAs. Especially in cancer, there is plenty of RNA-sequencing data to interrogate in vivo expression.

I would also like to see some recommendations related to unraveling the mechanism by which presumed functional lncRNAs operate. Establishing a phenotype upon knock-down is one thing, unravelling the mechanism can provide further support for function and relevance.

**Response to reviewers**

**Reviewer #1**

Gao and colleagues present a review of the current state of research into the functions of lncRNAs, and particularly into how the various perturbation tools should be interpreted. The review is certainly timely and addresses a question of major importance. My comments are relatively minor.

***Response****: We wish to thank the reviewer for the kind words and for the very helpful comments below! We modified the text according to the reviewer’s suggestions and highlighted the modified parts in yellow.*

- Antisense oligonucleotides are more often referred to as "ASO" not "AO"

***Response****: “AO” has been changed to “ASO” throughout the revised manuscript.*

- It should be mentioned that siRNAs often give different results to ASOs for the same target, and it has been hypothesised that this may be due to the fact that siRNAs are targeting cytoplasmic populations of the lncRNA, whereas ASOs get the nuclear population. See for example Maamar et al PMID 23723417

***Response****: A good suggestion. We added this discussion to the revised manuscript (Page 22, paragraph 2).*

*“However, caution should also be exercised when interpreting inconsistent results from different experimental approaches because each reverse-genetics method might target unique pools of transcripts derived from the same locus. For example, while both ASO- and RNAi-mediated knockdowns resulted in similar levels of depletion of lncRNA linc-HOXA1, only the former method affected a specific subset of linc-HOXA1 RNA molecules – those associated with sites of transcription – and led to the cis phenotype of suppression of nearby Hoxa1 gene expression.”*

- I found the section 'Evidence supporting the biological roles of lncRNAs" a little formulaic, citing various studies one after the other, without enough synthesis and interpretation of the overall results.

***Response****: We modified the section to make it flow better and to improve the logical connections between the various cited studies.*

- Section "LncRNAs appear to be dispensable for a vertebrate organism". While I do agree with the arguments put forth, the authors in my view do not give enough weight to data arguing against these findings. There are numerous papers on protein coding genes that yield no phenotype, or extremely subtle phenotypes such as FOXP2. There are papers showing phenotypic effects of knockout of ultraconserved elements. There are papers showing that lncRNA phenotypes only become apparent in more natural field trials (<https://doi.org/10.1016/j.bbr.2004.02.015>). The final criterion of functionality is in the survival fitness of animals in the wild - which is far from being modelled in laboratory conditions. This is the most likely explanation for extremely highly conserved and abundant RNAs like MALAT1 or NEAT1, which yield such subtle phenotypes in knockout animals.

***Response****: The reviewer again makes very good points. We agree that the laboratory conditions have obvious limitations since many phenotypes may only appear under specific environmental conditions that can only be modelled in natural environment. We included the relevant discussion of the BC1 semi-natural phenotypic studies pointed out by the reviewer into the section “Can lncRNAs still have biological functions” under the subsection of “Missed phenotype” (Page 20, paragraph 2). Furthermore, in this subsection we emphasized that “Even in the protein-coding gene realm, it is common for a knockout animal to have either no observable phenotype or phenotype revealed only under certain environmental or genetic conditions” (Page 20, paragraph 3).*

- In general I think the authors pay too little attention to evidence for lncRNA functionality from evolutionary sources. There is clear evidence from Haerty+Ponting, Ulitsky, Stadler (<https://www.ncbi.nlm.nih.gov/pmc/articles/PMC4408788/>) that lncRNAs are under purifying evolutionary selection. Such selection would be hard to reconcile if lncRNAs did not play roles in fitness.

***Response****: The reviewer is correct and we remedied this issue by including discussion of evolutionary conservation support for lncRNA functionality in the new subsection “Evolutionary conservation” in the section “Evidence supporting the biological roles of lncRNAs” (Page 5, paragraph 2, 3).*

-Table 1: I found this a very useful resource, since I'm not aware of any table of lncRNA knockout animals. I would encourage the authors to make this more comprehensive to cover all the present mouse knockouts, which I understand only amount to a handful at present.

***Response****: According to the suggestion of the reviewer, we expanded Table 1 to include not only studies about the “gold standard lncRNAs”, but also to our knowledge most of the mouse knockout studies of the other lncRNAs. As the result, the table was expanded from 11 to 87 in vivo phenotypic studies. With the exception of one study, we focused on works performed in mouse. The text part was also modified accordingly.*

- Page12: I don't find it helpful to so extensively discuss your own unpublished paper, since the author is unable to read it. This could be remedied by submission to Biorxiv at least.

***Response****: The unpublished manuscript is currently under advanced stages of review elsewhere. We are not certain whether submitting it into Biorxiv at this stage would conflict with a specific policy of the journal. We are attaching a preprint of this work in the file “Xu_etal_preprint.pdf” and if hopefully, it is accepted soon, we could add a reference to this review. If not, we can delete the references to our unpublished work from this review.*

- Evidence from C elegans: Another recent paper demonstrating functional lncRNAs in worm:[https://www.ncbi.nlm.nih.gov/pubmed/30777050](https://www.ncbi.nlm.nih.gov/pubmed/30777050)

***Response****: This example has been put into the revised manuscript (Page 4, paragraph 1).*

**Reviewer #2**

Gao and colleagues have produced a timely review on lncRNA functionality by bringing together studies at both end of the spectrum. Their manuscript provides a great overview of the potential issues associated with individual genetic approaches aimed at perturbing lncRNA expression or function. They also provide several suggestions on how to approach the problem. I only have a couple of minor comments that are worthwhile considering.

***Response****: We wish to thank the reviewer for the kind words and for the very helpful comments below! We modified the text according to the reviewer’s suggestions and highlighted the modified parts in yellow.*

The lncRNA database lncipedia contains > 1000 papers describing functions of individual lncRNAs. They authors may also refer to this when discussing literature support for lncRNA functionality.

***Response****: A good suggestion. We added references to LNCipedia as well as other databases containing information on functional annotations of lncRNA to the revised manuscript (Page 3, paragraph 1).*

*“Annotation and functional characterization status of a particular lncRNA could be obtained by querying manually-curated databases such as Lnc2Cancer, LncRNADisease, or LNCipedia”*

On page 7, when discussing off-target effects of siRNAs/AOs, the authors may which to add that most studies use multiple siRNAs/AOs against the same target lncRNA to demonstrate on-target effects and cancel out off-target effect. They could also make suggestions to the readership that, when working with these strategies, using multiple siRNA/AOs is a must.

***Response****: Another very good point that we addressed by including the relevant text into the revised manuscript (Page 17, paragraph 2).*

On page 14, the authors state that many lncRNAs have been found in cell lines and in vivo expression patterns are not known. While we are clearly missing information on the spatio-temporal expression, we do have access to in vivo expression datasets like the ones generated by TCGA, GTEX, FANTOM, … that do provide in vivo expression of lncRNAs. Especially in cancer, there is plenty of RNA-sequencing data to interrogate in vivo expression.

***Response****: The reviewer is correct and we have included the references to ENCODE (human and mouse), TCGA, GTEX and FANTOM5 into the revised manuscript (Page 23, paragraph 2).*

I would also like to see some recommendations related to unraveling the mechanism by which presumed functional lncRNAs operate. Establishing a phenotype upon knock-down is one thing, unravelling the mechanism can provide further support for function and relevance.

***Response****: This is of course a very relevant topic, however, it would be quite hard to develop it fully in this review given the space constraints. To address this, we have provided references to 9 recent reviews that delve into details of this subject. We hope that the reviewer finds this satisfactory (Page 2, paragraph 2).*
